# Pediatric obsessive–compulsive disorder as a developmental disorder of cognitive–emotional control: a transdiagnostic and family-integrated perspective

**DOI:** 10.3389/fpsyt.2025.1750938

**Published:** 2026-01-16

**Authors:** Cristina Di Vincenzo, Francesco Demaria, Ilaria Bertoncini, Deny Menghini, Alessandro Antonietti, Stefano Vicari, Maria Pontillo

**Affiliations:** 1 Department of Psychology, Catholic University of Sacred Hearth, Milan, Italy; 2 Child and Adolescent Neuropsychiatry Unit, Bambino Gesù Children’s Hospital, Istituto di Ricovero e Cura a Carattere Scientifico/Scientific Institute for Research, Hospitalization and Healthcare (IRCCS), Rome, Italy; 3 Department of Life Sciences and Public Health, Catholic University of the Sacred Hearth, Rome, Italy

**Keywords:** cognitive–emotional control, family accommodation, obsessive–compulsive disorder, pediatric OCD, transdiagnostic approach

## Abstract

Pediatric obsessive–compulsive disorder (OCD) is a complex condition that typically emerges in childhood or adolescence and is closely linked to developmental changes in cognitive and emotional control. This mini-review offers a clinically oriented synthesis of pediatric OCD from a developmental and transdiagnostic perspective, framing it as a disturbance of flexibility, inhibition, and distress regulation that organizes its clinical presentation. Variations in these control processes across development shape the content and form of obsessive–compulsive symptoms and contribute to their frequent overlap with conditions such as autism spectrum disorder, tic disorders and Tourettic OCD, ADHD, bipolar disorder, and psychosis-risk presentations, which share similar regulatory vulnerabilities. Within this framework, family accommodation is conceptualized as an interpersonal extension of the child’s regulatory difficulties, temporarily reducing distress while reinforcing reliance on external control. A transdiagnostic focus on underlying regulatory mechanisms also helps to clarify why interventions such as developmentally adapted cognitive-behavioral therapy with exposure and response prevention, family-focused treatments, and process-based transdiagnostic protocols can promote more flexible cognitive–emotional regulation in both the child and the family system. Taken together, these elements support a developmental, family-integrated, and transdiagnostic conceptualization of pediatric OCD centered on cognitive–emotional control.

## 1 Introduction

Obsessive–compulsive disorder (OCD) is a neurobehavioral condition characterized by the presence of obsessions, intrusive, recurrent thoughts perceived as involuntary and beyond the individual’s control, and compulsions, which manifest as repetitive behaviors or mental acts aimed at reducing the distress triggered by such thoughts (1). These symptoms can cause significant impairment in daily functioning and quality of life. The global prevalence of OCD in children and adolescents is estimated at approximately 0.8–1%, with a mean age of onset around 10 years (2). However, underdiagnosis remains common, as many children do not receive timely or adequate assessment and treatment; the duration of untreated OCD remains among the longest of psychiatric disorders, with diagnostic delays averaging more than two years in pediatric samples (3). More than half of adult OCD cases have their onset during childhood or adolescence (4), supporting the disorder’s developmental continuity, although a subset of patients may achieve partial remission over time (5). Since the DSM-5-TR, OCD has been classified as distinct from anxiety disorders, within the broader category of obsessive–compulsive and related disorders (6). This reclassification reflects growing evidence that, although anxiety is a frequent component, it is not the defining feature of OCD. The disorder is instead marked by intrusive, ego-dystonic thoughts and compulsive behaviors that are phenomenologically and neurobiologically distinct from the fear and avoidance typical of anxiety disorders. Neuroimaging meta-analyses highlight altered fronto-striatal connectivity and hyperactivation in cortico-striato-thalamo-cortical networks, contrasting with the amygdala-centered dysregulation observed in anxiety (7). Moreover, OCD displays a distinct cognitive profile, characterized by impairments in executive control, response inhibition, and cognitive flexibility—core domains of self-regulation and top-down modulation of emotion (8). Clinical and epidemiological data summarized in recent reviews support the affinity of OCD with other obsessive–compulsive spectrum disorders, including body dysmorphic disorder and trichotillomania (9). Within this spectrum, a tic-related OCD subtype, often termed “Tourettic OCD”, has been increasingly recognized, particularly in developmental samples (10). Conversely, clinical presentations characterized primarily by hoarding symptoms have been separated into a distinct condition.

**Figure 1:**
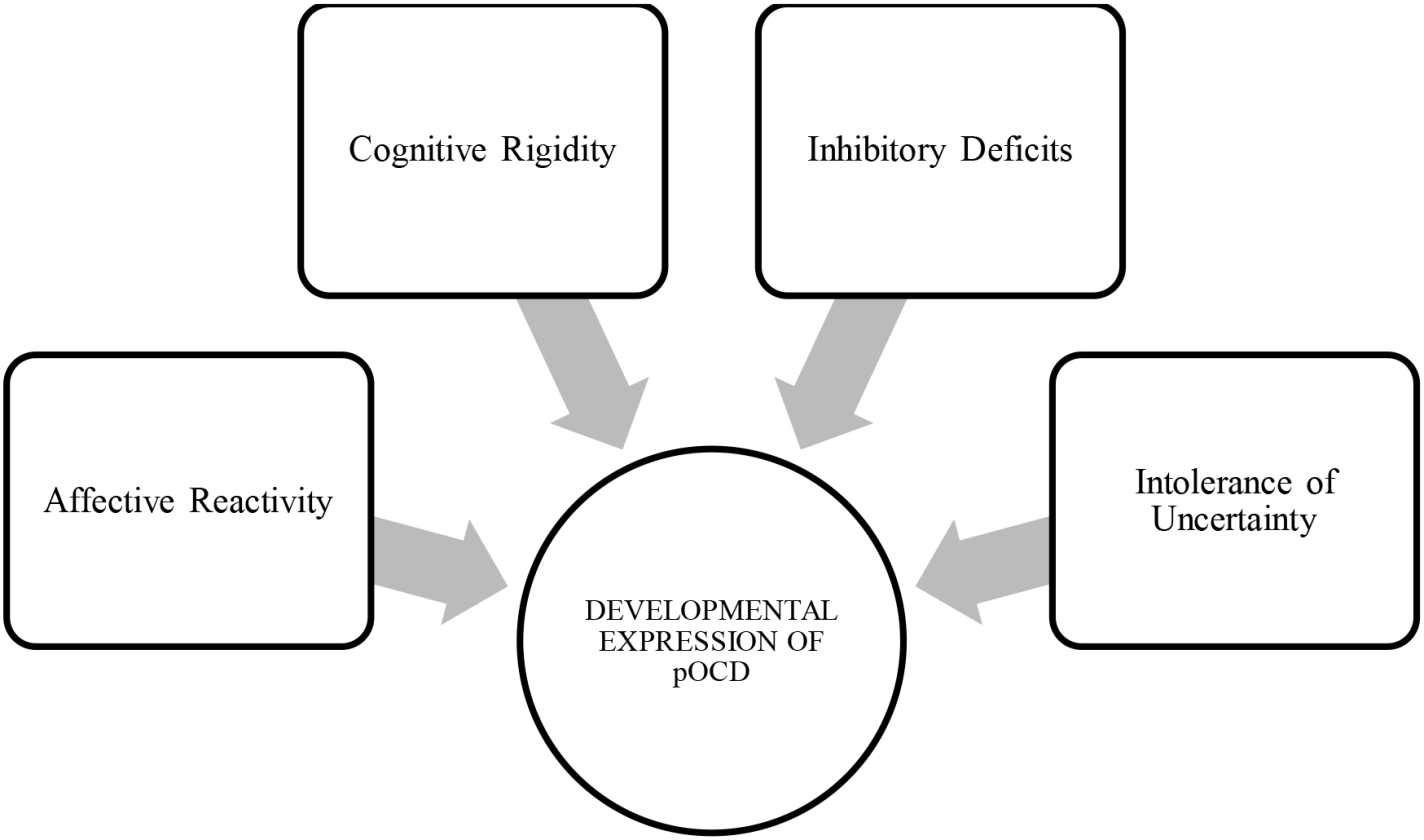
Transdiagnostic prospective of pediatric OCD.

Beyond its categorical definition, emerging developmental and transdiagnostic perspectives suggest that pediatric OCD may be best conceptualized as a disorder of cognitive and emotional control, characterized by rigidity, impaired cognitive flexibility, and difficulties managing uncertainty and threat across development (see **Figure 1**) (11–13). Within this framework, emotional control refers to the set of processes that enable the child to modulate emotional arousal, distress responses, and threat-related cognitions in a flexible, goal-directed manner across development. Recent work on pediatric OCD and related emotional disorders has highlighted that difficulties in emotion regulation, heightened distress reactivity, and reduced tolerance for uncertainty represent central regulatory vulnerabilities in youth with OCD (13, 21, 73). Converging evidence from cognitive neuroscience and developmental psychopathology indicates that these deficits in executive and regulatory control contribute to the persistence and phenomenology of obsessive–compulsive symptoms in youth (11–13). These self-regulatory impairments interact with familial processes, such as parental anxiety, accommodation, and emotion-regulation styles, which may temporarily reduce distress but inadvertently maintain compulsive cycles and reinforce maladaptive coping patterns (14). Family accommodation in particular has been consistently associated with greater symptom severity and poorer treatment response, highlighting the reciprocal nature of child and parent regulation in OCD (15).

This integrative framework situates pediatric OCD within a broader network of shared neurodevelopmental, affective, and family-based mechanisms that transcend traditional diagnostic boundaries, aligning with transdiagnostic models emphasizing common processes such as emotion dysregulation, cognitive inflexibility, and intolerance of uncertainty (16, 17). From this perspective, pediatric OCD exemplifies a developmental disorder of control that bridges the domains of cognition, emotion, and interpersonal regulation.

The present mini-review aims to provide a multidimensional synthesis of pediatric OCD through this integrative lens. Specifically, it examines phenomenology, comorbidity, family dynamics, and treatment approaches to illustrate how deficits in cognitive-emotional control and family modulation jointly shape the developmental course and clinical management of OCD in youth.

## 2 OCD across development: linking symptom dimensions to cognitive–emotional regulation and family involvement

### 2.1 Symptom dimensions across development

While OCD is characterized by a range of symptom dimensions, its clinical presentation may vary significantly across different developmental stages. Nevertheless, the core diagnostic features of OCD remain stable across the lifespan, and recent evidence supports the comparability of its latent dimensional structure from childhood through adulthood (2, 18, 19). The phenomenological nature of obsessions and compulsions is shaped by various factors, including age, cognitive development, and environmental context. A widely recognized characterization of obsessive-compulsive symptom dimensions, applicable across developmental stages, was proposed by Abramowitz et al. (20) (see **Table 1**). They described OCD symptoms as clustering into distinct but overlapping dimensions based on the content of obsessions and related compulsions. These include concerns about contamination and cleaning, fears of causing harm and compulsive checking, intrusive unacceptable thoughts (such as sexual, violent, or blasphemous content), a need for symmetry and difficulties discarding items associated with hoarding.

**Table 1:** Primary obsessions reported by 145 patients with OCD.

Category	Description
Contamination	Thoughts or mental images related to the doubt of having come into contact, even indirectly, with substances considered dangerous or disgusting
Harm	Thoughts or mental images related to potential harm to oneself or others - whether material, financial, or emotional- resulting from one’s own inattention, carelessness, or omissions
Superstitious	Thoughts or mental images related to negative events that may happen to oneself or others in the future if certain behavioral rules are not followed or if their negative effect is not neutralized
Somatics	Excessive preoccupation with parts of the body or physical appearance (dysmorphophobia)
Aggressive	Thoughts, mental images, or impulses to harm loved ones or oneself, despite not wanting to do so
Homosexuals	Thoughts, mental images, fantasies, or impulses with homoerotic content, which induce in the heterosexual individual the doubt of being a latent homosexual
Relational	Doubts about the adequacy of one’s partner and one’s feelings toward them; thoughts, mental images, fantasies, or sexual impulses toward individuals other than one’s partner, which trigger in the individual the obsessive doubt of not being in love with their partner
Religious or moral	Thoughts or mental images with blasphemous content, such as profanities, insults toward the deceased, sexualized fantasies toward sacred images, etc.
Sexual and pedophilic	Thoughts, mental images, fantasies, or sexual impulses toward inappropriate individuals such as relatives, animals, children, etc
Order and simmetry	Need for symmetry, uniformity or exactness. A sense that something isn’t done in the “right way”

Adapted from Abramowitz et al., 2006.

From a developmental perspective, these symptom dimensions may reflect different expressions of disrupted cognitive and emotional control: for example, checking and reassurance behaviors can be viewed as attempts to re-establish a sense of cognitive certainty in the face of intolerance of uncertainty (21); ordering and symmetry compulsions are often associated with cognitive inflexibility (impaired set-shifting) and ‘not-just-right’ experiences (22); whereas contamination-related concerns are often accompanied by heightened fear/disgust reactivity and difficulties modulating these affective states under threat, in line with emotion-regulation accounts of pediatric OCD (13, 23).

### 2.2 Developmental patterns in pediatric OCD

Normative rituals and routines exist along a continuum with obsessive–compulsive symptoms (24). In early childhood, behaviors such as bedtime routines, a preference for symmetry, or other structured activities often serve adaptive self-regulatory functions, offering predictability and comfort during daily transitions (25, 26). These behaviors frequently serve adaptive purposes, offering predictability and comfort. When children experience marked distress if these routines are not followed exactly or feel compelled to repeat actions until they feel ‘just right’ (e.g., brushing teeth), such behaviors may reflect emerging compulsions rooted in immature control systems (24).

Similarly, developmentally normative fears—such as concerns about monsters or ghosts—may become clinically significant when they persist, cause impairment, and lead to ritualistic behaviors like repeated checking or avoidance (27, 28). Obsessions in younger children often center on catastrophic fears, for instance, fears of harm or loss of attachment figures during separations from parents (2, 29) and compulsions in this age group tend to involve caregivers more frequently than in older populations (30, 31), highlighting the early interdependence between child self-regulation and parental co-regulation (32).

During adolescence, symptom content often shifts toward themes of sexuality, morality, and religion (2, 29). These obsessions reflect the increasing cognitive complexity and emotional introspection of adolescence, as youth engage in abstract moral reasoning, identity formation, and heightened sensitivity to social evaluation (2, 33, 34). Compulsions at this stage may function as maladaptive strategies to regulate internal conflict, guilt, or uncertainty, consistent with maturational changes in prefrontal–striatal circuitry supporting higher-order control (35–37). In adulthood, symptom profiles tend to consolidate into more stable and recognizable patterns, most commonly contamination fears and checking behaviors—with a decline in family involvement (2) (see **Table 2**). This progression parallels the increasing autonomy of self-regulation and the reduced external modulation by caregivers.

**Table 2:** Comparison of pediatric and adult-onset OCD (Geller et al., 2021).

Areas of investigation	Pediatric OCD	Adult-onset OCD
Prevalence	0.84% prevalence (1/3–1/2 remission rate)	1–3% prevalence
Age of onset	9–10 (with an SD of 2.5 years)	22–24 years
Gender ratio	F > M	F > M
OCD symptoms	Children-Intrusive fears of harm or loss of attachment figures. Hoarding. Symmetry and ‘just right’ phenomena. Fewer concrete cognitive obsessions. Adolescents-sexual, moral and religious themes, scrupulosity. Contamination fears.	Contamination, more stable over time and across fewer categories of obsessions/compulsion types.
Insight	Limited- only 63% have good or excellent insight	13.8–30.7% have poor to no insight
Comorbidity	Up to 80%- Mood and anxiety conditions, ADHD, Tic disorders, ODD, DMDD, ASD (~5%)	Mood and anxiety disorders
Family Role	Greater family involvement leads to worse OCD symptoms and greater functional impairment	Family accommodation also seen in relatives of adult-onset OCD but less direct involvement in rituals
Genetics	26% risk of OCD in a first degree relative	12% risk of OCD in a first degree relative
Adverse perinatal risk factors	Increased rates, especially in boys with OCD	Associated with an earlier age of OCD onset
Psychosocial stress	Increased rate of traumatic and stressful life events	Association with PTSD
Course and Outcome	Worse outcomes with co-morbid externalizing conditions and greater degrees of family accommodation. Overall higher rates of remission and symptoms becoming subclinical	Few cases of full remission over time

ASD, Autism Spectrum Disorder; ADHD, Attention-Deficit/Hyperactivity Disorder; ODD, Oppositional Defiant Disorder; DMDD, Disruptive Mood Dysregulation Disorder; PTSD, Posttraumatic Stress Disorder.

### 2.3 Assessment of symptoms, functional impairment, and family accommodation

Given the heterogeneity of clinical presentations and the developmental nuances of obsessive-compulsive symptoms, accurate and developmentally sensitive assessment is essential for early identification and differential diagnosis. Proper assessment tools provide valuable insights into severity, functional impairment, and family involvement.

Although the literature on assessment tools for pediatric OCD is less extensive than that for adults, several reliable and validated instruments are available for use with children and adolescents. Among the most widely used is the Children’s Yale-Brown Obsessive Compulsive Scale (CY-BOCS) (27), a semi-structured clinician-administered interview that evaluates the severity of obsessive and compulsive symptoms. Its revised version (CY-BOCS-II) (38) incorporates updates in OCD phenomenology and improves measurement precision. In addition to clinician-rated tools, self-report measures such as the Children’s Florida Obsessive-Compulsive Inventory (C-FOCI) (39) and the Obsessive Compulsive Inventory–Child Version (OCI-CV) (40) are commonly used to assess symptom presence and severity across different dimensions of OCD.

Other instruments have been developed to capture specific aspects of the disorder, including the Child Obsessional Compulsive Inventory–Revised (CHOCI-R) (41) for severity, the Child Obsessive-Compulsive Impact Scale–Revised (COIS-R) (42) for functional impairment, the Family Accommodation Scale–Self-Rated Version (FAS-SR) (43), and the OCD Family Functioning Scale (OFF) (44). From a developmental and process-based standpoint, these tools primarily quantify symptom burden, functional impact, and patterns of family involvement. They do not directly assess emotional-control mechanisms, but specific patterns captured by these instruments (e.g., higher symptom severity, avoidance-related impairment, or reliance on parental accommodation) can be interpreted clinically as indirect indicators of regulatory difficulties and reliance on external co-regulation. Although OCD-specific questionnaires do not measure emotional control directly, the broader developmental psychopathology literature has operationalized emotion-regulation abilities through validated instruments such as the Emotion Regulation Checklist (ERC) (45) and the Difficulties in Emotion Regulation Scale (DERS) (46) Gratz & Roemer, 2004), including applications in adolescent samples (47). While these emotion-regulation measures are not routinely employed in the assessment of pediatric OCD, they provide well-established frameworks for quantifying regulatory capacities—including modulation of arousal, affective lability, and the flexible use of regulatory strategies—which help clarify what is meant by “emotional control” in the present conceptual model. In particular, tools like the FAS-SR and the OFF help show how family dynamics are connected to symptom patterns (43, 44). Overall, mapping symptom dimensions across development while considering indirect clinical indicators of emotional control and family co-regulation provides a coherent lens for assessment and for tailoring mechanism-focused interventions.

## 3 Comorbidities as windows on cognitive–emotional control in pediatric OCD

Comorbidity is highly prevalent in pediatric OCD, with a meta-analysis estimating that 64% of affected youth meet criteria for at least one additional disorder (48). From a developmental–mechanistic perspective, comorbidities are not merely additive but illuminate distinct dimensions of control—from flexibility and inhibition to affective and sensory regulation—offering a transdiagnostic window into shared neurodevelopmental vulnerabilities. Broadly, these can be grouped into neurodevelopmental and affective–psychiatric domains.

### 3.1 Autism spectrum disorder

A substantial but heterogeneous overlap exists between ASD and OCD, with OCD prevalence in autistic samples ranging from 4–9% in community studies to 37% in clinical cohorts, and a meta-analytic estimate of 17% meeting OCD criteria. Longitudinal data indicate that autistic youth are more than twice as likely to later develop OCD, while children with OCD show an approximately fourfold increased likelihood of receiving an ASD diagnosis, with about 25% meeting ASD criteria (49).

Although both conditions share repetitive behaviors, their functions diverge: ASD is characterized by rule-bound, intrinsically motivated behaviors linked to restricted interests, whereas OCD compulsions aim to reduce anxiety and neutralize intrusive thoughts. Neurocognitive findings point to overlapping fronto-striatal circuits, yet different motivational drives—self-regulatory in ASD, anxiety-driven in OCD (50, 51).

The ASD–OCD overlap highlights shared difficulties in cognitive flexibility and habit formation, underscoring rigidity as a core transdiagnostic vulnerability across neurodevelopmental spectra.

### 3.2 Tic disorders and Tourettic OCD

OCD co-occurs in 30–60% of children with Tourette Syndrome (TS), and TS in 10–15% of youth with OCD (52). Both disorders involve repetitive behaviors preceded by inner tension—premonitory urges in TS and anxiety-laden discomfort in OCD—reflecting partially overlapping cortico-striato-thalamo-cortical (CSTC) circuits.

At their interface lies the Tourettic OCD (TOCD) phenotype, characterized by complex motor sequences performed until they feel “just right,” often driven by sensory or somatic urges rather than cognitive intrusions (10). These presentations tend to follow a more severe and treatment-resistant course, frequently requiring integrated approaches such as ERP for compulsive elements and CBIT for motor–sensory urges.

TS–OCD comorbidity illustrates the motor and sensory dimensions of control dysregulation, where failures in inhibitory gating and urge modulation blur the line between voluntary and involuntary acts.

### 3.3 Attention-deficit/hyperactivity disorder

Between 6–11% of youth with ADHD meet criteria for OCD, and an additional 10–12% exhibit subclinical obsessive–compulsive features (53). The co-occurrence reflects shared disruptions in fronto-striatal networks involved in response inhibition, attentional control, and goal-directed behavior.

Although impulsivity and compulsivity have traditionally been viewed as opposing tendencies, they may represent complementary expressions of impaired top-down control within a unified regulatory framework (54). In some cases, compulsions can serve compensatory functions, emerging as attempts to restore cognitive order in the face of executive disorganization.

The ADHD–OCD interface exemplifies the impulsivity–compulsivity continuum, linking disinhibition and overcontrol as opposite poles of disrupted cognitive regulation.

### 3.4 Bipolar disorder

OCD–BD comorbidity is increasingly recognized in youth, with prevalence estimates ranging from 15–40% and bidirectional overlap between the two disorders (55, 56). In pediatric samples, obsessive–compulsive symptoms often follow an episodic course, worsening during depressive or mixed states and remitting during (hypo)mania.

Phenomenologically, this comorbidity features existential, sexual, and aggressive obsessions, as well as hoarding behaviors, patterns consistent with fronto-limbic dysregulation and affective instability (57). Clinically, these youth present with greater functional impairment and suicidality, requiring careful mood stabilization before implementing ERP or SSRI-based interventions.

OCD–BD comorbidity underscores the affective regulation component of control dysfunction, where fluctuations in prefrontal–limbic balance modulate the intensity and function of compulsive strategies.

### 3.5 Psychosis spectrum and clinical high risk

In a large multicenter study of youth aged 10–17 at clinical high risk for psychosis (CHR-P), 50% exhibited clinically significant obsessive–compulsive symptoms (OCS), associated with greater prodromal severity and poorer functioning (58). Among pediatric OCD samples, UHR/basic-symptom criteria occur in approximately 43–44%, correlating with earlier onset, reduced insight, and greater impairment (59, 60).

Neurobiologically, both OCD and CHR-P populations show corticostriatal and anterior cingulate abnormalities, suggesting shared disruptions in salience processing, cognitive rigidity, and emotional regulation (60, 61).

Early obsessive–compulsive phenomena may therefore represent developmental variants of disturbed cognitive–emotional control mechanisms shared with the psychosis spectrum, supporting a continuum view of vulnerability expression.

**Figure 2:**
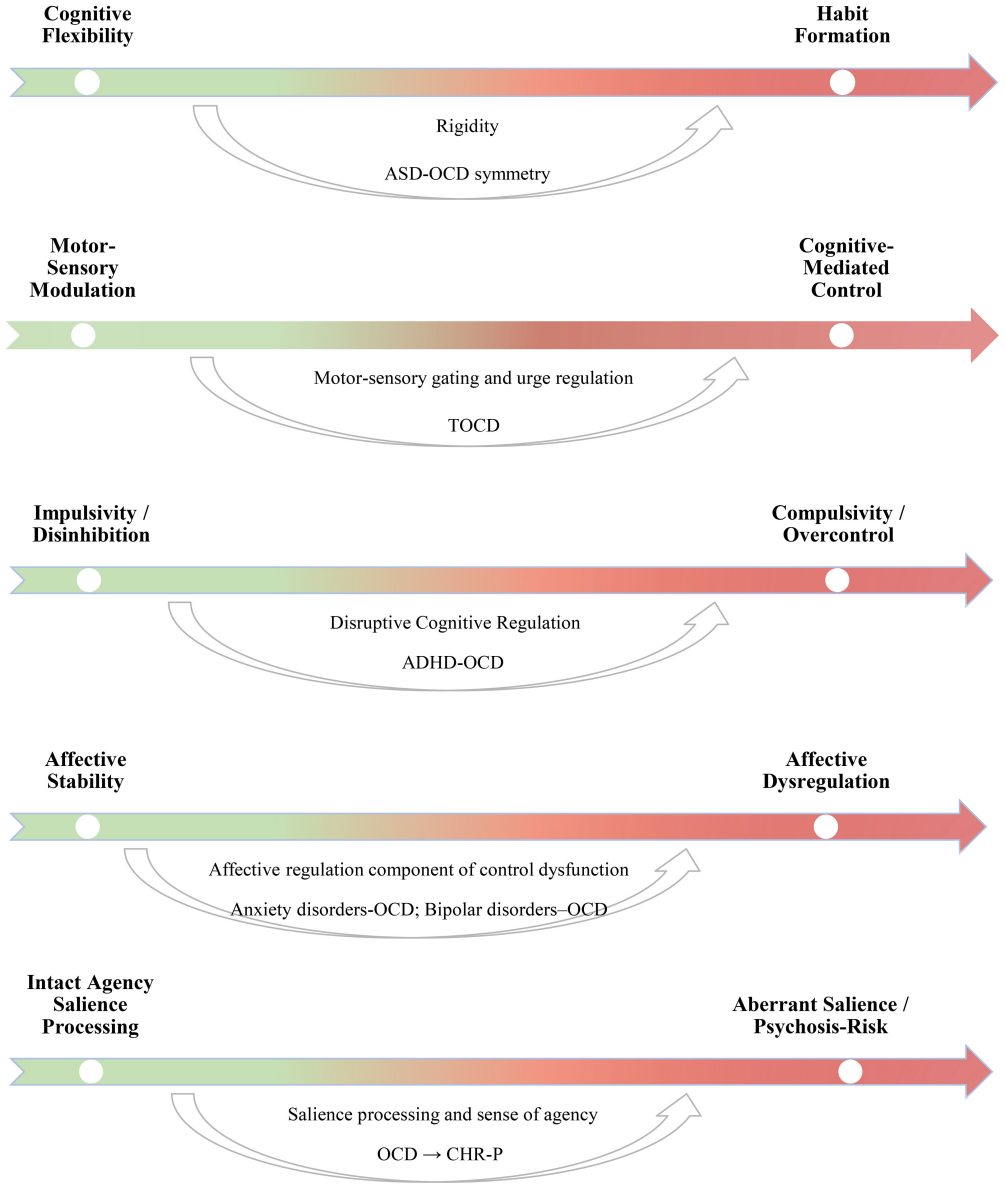
Transdiagnostic control dimensions in pediatric OCD comorbidity. These continua represent conceptual transdiagnostic dimensions of cognitive–emotional control. Individual children do not fall at the extremes of these continua; rather, they occupy different positions along each dimension and may show some, but not all, of them.

Taken together, comorbidities in youth OCD converge on a common profile of impaired control systems (flexibility, inhibition, emotion regulation) that emerge and reorganize developmentally. This pattern supports a transdiagnostic account in which pediatric OCD is understood as a developmental phenotype of disturbed cognitive–emotional control, expressed differently across neurodevelopmental and affective conditions. In **Figure 2**, we present our proposed conceptualization.

## 4 The role of family accommodation in pediatric OCD: a mechanism of external co-regulation and symptom maintenance

Beyond individual-level vulnerabilities, these regulatory difficulties extend to the interpersonal system, where family dynamics play a critical role in maintaining or alleviating symptoms.

Family accommodation (FA) is not a primary cause of obsessive–compulsive disorder, but it reliably functions as a mechanism of external co-regulation in pediatric OCD: by assisting with rituals, providing reassurance, or avoiding triggers, caregivers reduce the child’s distress in the short term while inadvertently maintaining obsessive–compulsive cycles over time (62–65). Within this framework, FA can be understood as a dyadic regulatory loop—the child’s anxiety shapes the parent’s behavior and the parent’s accommodation shapes the child’s reliance on external control—thereby constraining opportunities for autonomous coping, corrective exposure, and learning.

Empirically, higher FA is consistently associated with greater OCD severity (especially contamination/washing), increased internalizing (anxiety, depression) and externalizing symptoms (e.g., ADHD/ODD), and poorer global, social, and role functioning independent of anxiety or cognitive level (63, 64, 66–68). FA is also linked to lower self-esteem and perceived efficacy in youth, suggesting interference with the maturation of self-regulatory capacity (68). Caregivers with elevated anxiety sensitivity or a family history of anxiety appear more prone to accommodating responses, indicating that parental emotion-regulation styles can amplify the child’s control dysregulation (63).

These observations have clear treatment implications. Family-inclusive protocols that explicitly target accommodation, such as the structured four-phase model (alliance/psychoeducation, identification and graded reduction of accommodation, ERP coaching) proposed by Demaria et al. (69) (see **Table 3**)—aim to shift co-regulation from accommodation to supportive non-avoidance, restoring conditions under which exposure and response prevention (ERP) can be effective. Likewise, SPACE (Supportive Parenting for Anxious Childhood Emotions) trains caregivers to respond with support rather than accommodation, by mapping key accommodating behaviors, implementing planned reductions, and managing child distress while reinforcing competence and independence (70). Viewed through a developmental, process-based lens, family accommodation is best conceptualized as external co-regulation that alleviates anxiety in the short term but sustains symptoms over time (15, 71, 72), a relational expression of impaired cognitive–emotional control in youth OCD (13) aligns with a transdiagnostic, mechanism-focused formulation of the disorder.

**Table 3:** Psychoeducation intervention for parents of children and adolescents with OCD.

Session	Objective	Cognitive-behavioral key strategies
Session 1–2 THERAPEUTIC ALLIANCE	To build a therapeutic relationship between the psychotherapist and the parents	− Build a relaxed atmosphere − Investigate and inform of child’s strengths − Focused on child’ skills and psychological resources
Session 3–5 EDUCATION	To investigate and modify parents’ possible misconceptions of OCD	− Give information about: cause of the disease, the prevalence in childhood and adolescence, examples of possible manifestations, the symptomatology, and therapeutic options − Communicate hope, optimism, welcoming and restructuring expectation about the OCD symptoms reduction − Attribute symptoms to the OCD itself and not to the child providing information about how to do not blame the child for symptoms − Provide information according to family’s conversational style, repeat and clarify concepts, applied to different situations, and illustrated from different perspectives
Session 6–10 FAMILY ACCOMMODATION MANAGEMENT	Help to parents: a. To recognize parent’s involvement in child’s symptomatology b. To manage child’s OCD symptoms more effectively in family’s daily routines	− Explain Family Accommodation mechanism (maintenance of the disorder) − Share useful behaviors to apply in everyday life teaching how to avoid getting involved in compulsive rituals − Use role-play techniques to show parents how to react to a child’s specific compulsive rituals
Session 11–12 ERP TREATMENT EDUCATION	To prepare and to teach parents the ERP treatment	− Psychoeducation about ERP Treatment − Inform adequately parents on the procedure during the specific exposure − Ensure that the parents will follow the psychotherapist’s indication

Based on Demaria et al. (2021).

Recognizing family accommodation as a relational form of control dysregulation also clarifies its place within the broader therapeutic landscape. Because obsessive–compulsive symptoms are embedded in both individual and interpersonal regulatory loops, effective intervention must target control processes across levels, within the child (cognitive flexibility, tolerance of uncertainty), within the family system (patterns of co-regulation and accommodation), and within developmental context (gradual autonomy and mastery). This perspective naturally transitions from understanding family mechanisms to designing treatments that restore adaptive regulation.

## 5 Restoring cognitive–emotional control: process-based and developmentally informed treatment approaches

International guidelines identify both pharmacological and psychological interventions as effective treatments for OCD, with cognitive-behavioral therapy (CBT) recognized as the gold-standard approach (73). Compared with medication alone, CBT produces more enduring change, lower relapse rates, and fewer side effects (74). Recent data indicate remission rates of 39.3% for CBT alone, 21.4% for SSRIs, and 53.6% for combined treatment (75). From a process-based perspective, CBT, particularly through ERP, can be understood as a means of recalibrating these same control processes that become dysregulated at both the individual and interpersonal levels.

From a process-based perspective, CBT, particularly through ERP, can be understood as a means of restoring flexible cognitive–emotional control. In adults, ERP has been shown to improve intolerance of uncertainty, cognitive rigidity, and maladaptive avoidance, mechanisms central to the persistence of obsessive–compulsive symptoms (76, 77). Consistent with this rationale, the Unified Protocols for Children and Adolescents (UP-C/A) have recently been evaluated for OCD, alongside anxiety and depression. The protocol explicitly trains emotion regulation and cognitive flexibility within a transdiagnostic, process-based framework. In a sample of 388 youth, OCD symptoms improved more slowly in the first half of treatment but caught up in the second half; youth with and without OCD showed comparable gains in anxiety, depression, and transdiagnostic targets (anxiety sensitivity, cognitive flexibility, distress tolerance). These data also suggest that introducing exposure earlier may confer additional benefit (78).

Although direct mechanistic evidence in pediatric populations is still limited, emerging developmental findings suggest that these same processes, rigidity, intolerance of uncertainty, and emotion dysregulation, play a central role in childhood OCD and may represent key therapeutic targets for age-adapted ERP (13, 79).

Within this framework, treatment is not merely symptom reduction but a gradual recalibration of maladaptive control processes, such as inflated responsibility, perfectionism, and intolerance of uncertainty, that maintain obsessive-compulsive symptoms. These cognitive shifts may also enhance emotion-regulation capacities, as emerging evidence suggests that emotion dysregulation plays a role in the maintenance and therapeutic change of pediatric OCD (13, 76, 80).

In children and adolescents, the developmental tailoring of CBT is essential to ensure emotional engagement and cognitive comprehension throughout the therapeutic process (81, 82). Early sessions typically focus on psychoeducation and the collaborative identification of feared situations and avoidance patterns, fostering a sense of agency and mastery. Subsequent stages target distorted control beliefs, such as overestimation of responsibility, perfectionism, or intolerance of uncertainty, before introducing ERP exercises that are adapted to the child’s developmental level and emotional maturity (81, 83, 84).Consistent with international recommendations, a stepped-care approach is increasingly endorsed to match treatment intensity to clinical severity. For youth with mild functional impairment, guided self-help combined with family psychoeducation may suffice, while those with moderate to severe symptoms require full CBT with ERP, caregiver involvement, and developmental adaptations (73). When psychological therapy is declined or not feasible, SSRIs remain an evidence-based adjunct, provided careful monitoring and integration with behavioral principles. While stepped-care models adjust treatment intensity according to clinical severity or response, staged-care approaches (85) organize interventions around underlying control mechanisms and developmental needs. Building on this principle, Farrell et al. (85) introduced a staged-care CBT–ERP model for pediatric OCD that operationalizes a mechanism-based hierarchy: interventions are scaled according to shared control mechanisms, such as anxiety, avoidance, and emotion dysregulation, and adjusted for developmental stage. This model bridges process-based theory and routine clinical practice, demonstrating how stepped delivery systems can translate mechanistic understanding into scalable care.

Yet, even mechanism-oriented approaches should be situated within the child’s relational environment, as family dynamics crucially shape both the onset and maintenance of obsessive-compulsive symptoms. Interventions that explicitly target family accommodation, such as the Supportive Parenting for Anxious Childhood Emotions (SPACE) program (70) or the structured four-phase family model (69), help parents shift from accommodating distress to supporting exposure-based coping, thereby restoring conditions under which ERP can be effective. Meta-analytic evidence indicates that broader family variables, including conflict, blame, overprotection, and poor cohesion, predict treatment resistance, underscoring the need for multidimensional, family-focused interventions (14).

Emerging third-wave approaches, such as Acceptance and Commitment Therapy (ACT), extend this process-based rationale by cultivating psychological flexibility, the capacity to engage with distressing internal experiences while pursuing valued goals (86–88). In younger children, behavioral and parent-mediated strategies remain preferable, focusing on reducing accommodation and scaffolding self-regulation capacities (89).

### 5.1 Integrating transdiagnostic processes into clinical care

To better support the translation of mechanisms into practice, we outline how a mechanistic and developmentally sensitive assessment contributes to an integrated treatment approach for pediatric OCD. Within this framework, the evaluation of symptoms is complemented by attention to transdiagnostic control processes—such as cognitive rigidity, inhibitory difficulties, and challenges in emotion regulation—as well as to developmental stage, family accommodation, and comorbid neurodevelopmental or affective features. Formulating the case along these dimensions does not replace standard diagnostic assessment, but provides a structure for understanding which regulatory mechanisms are most implicated and how they interact with the child’s relational and developmental context.

This type of formulation can help clinicians anticipate how established CBT/ERP components may need to be emphasized or adapted. For example, prominent rigidity or “not-just-right” experiences may signal the need for more carefully graded hierarchies, while difficulties modulating distress may suggest the utility of preliminary work on emotional awareness or caregiver-supported co-regulation to prepare for exposure. High levels of family accommodation, in turn, can indicate that parent-focused modules aimed at reducing accommodating behaviors may be important to implement alongside child-directed ERP. Rather than prescribing new interventions, these considerations highlight how mechanistic information can guide the organization and pacing of existing evidence-based approaches.

Comorbid features can further refine this planning. Autistic traits may inform the use of increased structure, predictable routines, and concrete language; tic-related or sensory-based urges may help identify aspects of compulsivity that can be explicitly incorporated into exposure tasks; attentional or mood-related difficulties may influence session structure or the timing of more demanding ERP elements. In each case, these features function not as separate treatment pathways but as contextual markers that can shape feasibility, engagement, and focus within standard CBT/ERP.

Finally, stepped or staged care models offer complementary frameworks for aligning assessment with intervention intensity. Stepped care allows clinical resources and treatment demands to be matched to functional impairment and adjusted over time, whereas staged care organizes interventions around core control mechanisms shared across diagnostic presentations. When combined, these approaches provide a coherent sequence, from mechanistic assessment to case formulation to individualized planning, that supports flexible, context-sensitive application of established treatments within the heterogeneous presentations of pediatric OCD.”

## 6 Limitations of categorical systems and emerging transdiagnostic perspectives in pediatric OCD/rethinking pediatric OCD through a transdiagnostic and developmental lens

Pediatric OCD can be conceptualized as a developmental disturbance of cognitive–emotional control, rather than as a discrete categorical condition. Over the past decade, research has moved beyond traditional diagnostic boundaries toward dimensional and transdiagnostic frameworks that situate obsessive–compulsive phenomena within broader developmental pathways of internalizing psychopathology. Conventional nosological systems such as DSM and ICD often fail to capture the fluidity and overlap of symptoms commonly observed in childhood and adolescence, where anxiety, depression, and compulsivity frequently co-occur and evolve dynamically over time.

From a developmental psychopathology perspective, OCD emerges from partially shared mechanisms, such as emotion dysregulation, intolerance of uncertainty, and cognitive inflexibility, that cut across diagnostic categories. Longitudinal studies show that early behavioral rigidity and anxious traits predict later obsessive–compulsive symptoms (90), suggesting that OCD may emerge along a broader continuum of maladaptive control and internalizing distress (91). This dimensional view does not negate the specificity of OCD but instead situates it within a broader framework of developmental vulnerabilities that interact over time.

Recent theoretical advances have reinforced this perspective. Garnaat et al. (92), applying the Research Domain Criteria (RDoC) to pediatric OCD, propose that dysfunctions in the Potential Threat and Habit Formation domains may explain the transition from anxiety-driven rituals to compulsive, automatized behaviors as fronto-striatal circuits mature. Similarly, dimensional frameworks such as HiTOP integrate obsessive–compulsive phenomena within the internalizing spectrum, reflecting shared neural substrates and affective processes. The EMERGE study (93) further supports this view by demonstrating that transdiagnostic mechanisms—such as intolerance of uncertainty, behavioral avoidance, and repetitive negative thinking—predict the co-development of obsessive–compulsive, depressive, and attenuated psychotic symptoms in adolescents. Taken together, these findings underscore the importance of targeting regulatory mechanisms, rather than categorical symptoms, in both research and clinical intervention.

From a clinical standpoint, this shift has practical advantages. Transdiagnostic and process-based interventions, such as UP-C/A, directly target regulatory dysfunctions underlying OCD and related emotional disorders. Empirical findings indicate that improvements in cognitive flexibility and distress tolerance mediate symptom reduction across anxiety, depression, and OCD (94). Focusing on these core transdiagnostic processes may optimize treatment outcomes and prevent symptom migration between diagnostic categories.

Importantly, conceptualizing OCD dimensionally also aligns with its known heterogeneity. Pediatric OCD often presents with fluctuating symptom profiles, high comorbidity with anxiety and tic disorders, and variable levels of insight. Dimensional models accommodate this variability by viewing compulsivity and intrusive thinking as expressions of broader regulatory and neurocognitive dysfunctions rather than discrete disease entities. They also highlight developmental timing—showing that early emotional rigidity, maladaptive habit learning, or difficulty inhibiting prepotent responses may constitute developmental risk markers for later OCD onset.

In sum, applying transdiagnostic and developmental models to pediatric OCD allows for a more integrated understanding of its onset, course, and treatment. By clarifying the regulatory mechanisms that underlie OCD and related disorders, these models refine its conceptualization and support early, mechanism-focused interventions aimed at strengthening cognitive flexibility, enhancing emotion regulation, and improving tolerance of uncertainty across development. Together, transdiagnostic and developmental frameworks converge on the view of pediatric OCD as a disorder of cognitive–emotional control. This integrative lens not only accounts for comorbidity and heterogeneity but also informs mechanism-focused and family-integrated interventions that target maladaptive control processes within the child’s relational context.

## 7 Conclusion

Viewing pediatric OCD as a disorder of cognitive–emotional control, modulated by family dynamics, offers a unifying framework that reconciles developmental trajectories, comorbidity patterns, and treatment mechanisms. This perspective bridges categorical diagnosis with transdiagnostic science, clarifying how rigidity, emotion dysregulation, and intolerance of uncertainty shape symptom expression across stages of development and within relational contexts. Clinically, it supports earlier, mechanism-focused, and context-sensitive care: developmentally adapted CBT/ERP, family-integrated approaches that reduce accommodation and scaffold regulation, and flexible stepped/staged delivery that matches interventions to shared control mechanisms. Research and services aligned with this lens, linking mechanistic assessment to outcomes and prioritizing scalable, family-informed models, can improve precision in evaluation and durability of treatment gains, ultimately advancing quality of life for children and adolescents with OCD.
